# Mining the Influencing Factors and Their Asymmetrical Effects of mHealth Sleep App User Satisfaction From Real-world User-Generated Reviews: Content Analysis and Topic Modeling

**DOI:** 10.2196/42856

**Published:** 2023-01-31

**Authors:** Mingfu Nuo, Shaojiang Zheng, Qinglian Wen, Hongjuan Fang, Tong Wang, Jun Liang, Hongbin Han, Jianbo Lei

**Affiliations:** 1 Institute of Medical Technology, Health Science Center, Peking University Beijing China; 2 Cancer Institute The First Affiliated Hospital of Hainan Medical University Haikou China; 3 Department of Pathology, Hainan Women and Children Medical Center, Hainan Medical University Haikou China; 4 Department of Oncology The Affiliated Hospital of Southwest Medical University Luzhou China; 5 Department of Endocrinology Beijing Tiantan Hospital Capital Medical University Beijing China; 6 Department of Medical Informatics, School of Public Health Jilin University Changchun China; 7 IT Center, Second Affiliated Hospital, School of Medicine, Zhejiang University Hangzhou China; 8 School of Public Health, Zhejiang University Hangzhou China; 9 Department of Radiology Peking University Third Hospital, Health Science Center Peking University Beijing China; 10 Clinical Research Center The Affiliated Hospital of Southwest Medical University Luzhou China; 11 Center for Medical Informatics Health Science Center Peking University Beijing China; 12 School of Medical Informatics and Engineering, Southwest Medical University Luzhou China

**Keywords:** sleep disorder, mobile health applications, topic modeling, Herzberg’s 2-factor theory, machine learning

## Abstract

**Background:**

Sleep disorders are a global challenge, affecting a quarter of the global population. Mobile health (mHealth) sleep apps are a potential solution, but 25% of users stop using them after a single use. User satisfaction had a significant impact on continued use intention.

**Objective:**

This China-US comparison study aimed to mine the topics discussed in user-generated reviews of mHealth sleep apps, assess the effects of the topics on user satisfaction and dissatisfaction with these apps, and provide suggestions for improving users’ intentions to continue using mHealth sleep apps.

**Methods:**

An unsupervised clustering technique was used to identify the topics discussed in user reviews of mHealth sleep apps. On the basis of the two-factor theory, the Tobit model was used to explore the effect of each topic on user satisfaction and dissatisfaction, and differences in the effects were analyzed using the Wald test.

**Results:**

A total of 488,071 user reviews of 10 mainstream sleep apps were collected, including 267,589 (54.8%) American user reviews and 220,482 (45.2%) Chinese user reviews. The user satisfaction rates of sleep apps were poor (China: 56.58% vs the United States: 45.87%). We identified 14 topics in the user-generated reviews for each country. In the Chinese data, 13 topics had a significant effect on the positive deviation (PD) and negative deviation (ND) of user satisfaction. The 2 variables (PD and ND) were defined by the difference between the user rating and the overall rating of the app in the app store. Among these topics, the app’s sound recording function (β=1.026; *P*=.004) had the largest positive effect on the PD of user satisfaction, and the topic with the largest positive effect on the ND of user satisfaction was the sleep improvement effect of the app (β=1.185; *P*<.001). In the American data, all 14 topics had a significant effect on the PD and ND of user satisfaction. Among these, the topic with the largest positive effect on the ND of user satisfaction was the app’s sleep promotion effect (β=1.389; *P*<.001), whereas the app’s sleep improvement effect (β=1.168; *P*<.001) had the largest positive effect on the PD of user satisfaction. The Wald test showed that there were significant differences in the PD and ND models of user satisfaction in both countries (all *P*<.05), indicating that the influencing factors of user satisfaction with mHealth sleep apps were asymmetrical. Using the China-US comparison, hygiene factors (ie, stability, compatibility, cost, and sleep monitoring function) and 2 motivation factors (ie, sleep suggestion function and sleep promotion effects) of sleep apps were identified.

**Conclusions:**

By distinguishing between the hygiene and motivation factors, the use of sleep apps in the real world can be effectively promoted.

## Introduction

### Background

Approximately a quarter of the global population has sleep problems [1-4], such as sleep deprivation, excessive sleep, and reduced sleep quality, resulting in health, safety, and economic issues. These sleep problems not only reduce the quality of life but also increase the risk of various diseases, such as high blood pressure [5], diabetes [6], and digestive diseases [7]. Sleep problems can also cause economic losses [8] and lead to traffic accidents [9]. However, in the context of the COVID-19 epidemic, traditional offline sleep interventions are difficult to perform. Therefore, a convenient, remote, and effective tool is necessary to solve or alleviate the global challenge of sleep problems.

Mobile health (mHealth) apps have the potential to provide this tool; however, their use in the real world is poor. The popularity of mobile phones and the immediacy and convenience of the mobile internet have led to the unique advantage of mHealth apps in providing sleep management services, and their effectiveness has been recognized in research. For example, Hasan et al [10] collected data from 54 randomized controlled trials, including 11,815 participants. They found that compared with conventional sleep interventions, cognitive behavioral therapy for insomnia–based apps significantly increased total sleep time, reduced sleep latency time, and improved sleep efficiency and quality. However, the real-world use of mHealth sleep apps is poor; nearly 25% of users stop using them after one use [11]. The existing literature indicates that user satisfaction is a major factor influencing continuous intention [12,13]. Therefore, exploring the factors that influence user satisfaction is vital for improving the real-world use of mHealth sleep apps.

Existing research on user satisfaction with mHealth sleep apps is limited by the small sample size, high costs, and difficulty in generalizing the findings. For example, Buman et al [14] used structured interviews to explore the acceptability and satisfaction of an mHealth sleep app (BeWell24) among 26 veterans. Similarly, Huberty et al [15] used interviews to explore the satisfaction and acceptability of an mHealth sleep app (Calm) among 88 college students. Philip et al [16] developed an mHealth app (KANOPEE) that supports the diagnosis of sleep disorders and interventions. Then, they explored the acceptability of the app by distributing questionnaires to 2000 users via the internet.

In the field of mHealth sleep apps, research based on real-world big data deeply exploring the factors influencing user satisfaction is lacking. Existing studies have shown that user satisfaction factors are asymmetrical. For example, the two-factor theory suggests that there is an asymmetrical effect between the factors influencing user satisfaction and dissatisfaction [17]. Influencing factors can be divided into motivation and hygiene factors. Motivation factors refer to the value-added attributes that users usually do not expect (ie, when some features of the app meet user expectations, users are satisfied, and when they do not meet expectations, users are not disappointed). Hygiene factors are basic attributes (ie, users will not feel satisfied when the feature of the app meets their expectations and are disappointed when the feature does not meet their expectations) [17]. Failure to consider the asymmetry of factors influencing user satisfaction may lead to poor construction and predictive power of the explanatory models [18].

Guided by the two-factor theory and using the internet as the data source to explore the factors influencing user satisfaction and dissatisfaction with mHealth sleep apps can, we can alleviate the above problems and fill the gaps in the research. Moreover, the number of user reviews is large and can reflect how the apps are used in the real world. Thus, the conclusions obtained may be more objective with less bias. Studies have analyzed user reviews of weight loss and precision nutrition apps [19,20]; however, research on mHealth sleep apps is lacking.

### Objectives

Therefore, in this study, we used machine learning–based topic modeling to analyze user reviews of 10 mainstream mHealth sleep apps in Chinese and American mobile app stores. This study had the following research objectives:

1. Mine and compare Chinese and American user viewpoints of mHealth sleep apps and explore the factors influencing user satisfaction and dissatisfaction with mHealth sleep apps.
2. Validate the asymmetry of influencing factors of mHealth sleep app on user satisfaction and dissatisfaction and identify motivation and hygiene factors of the mHealth sleep app.
3. Provide suggestions for improving users’ intentions to continue using mHealth sleep apps.

## Methods

### Data Collection

#### Identifying 10 Mainstream Apps

In March 2022, we conducted a comprehensive search of sleep-related mHealth apps across leading mobile app stores in China (Chinese Apple App Store, Huawei App Store, Xiaomi App Store, and VIVO App Store) and the United States (US Apple App Store and US Android Google Play Store). The following search terms were used for each app store: “sleep,” “sleep management,” “sleep monitoring,” and “sleep tracking.” After deduplication, we identified 131 unique apps in 4 Chinese mobile app stores and 193 unique apps in 2 US mobile app stores.

The inclusion criteria for the apps were as follows: (1) focus on sleep self-management based on user-generated data, (2) can be used without the assistance of health care givers, and (3) language in English or Chinese. We excluded the apps that met the following exclusion criteria: (1) cannot work properly; (2) app for special groups only, such as infants or the older adults; and (3) sleep management is not the main function or purpose of the app, for example, some comprehensive health management apps.

Finally, 5 unique apps with the highest number of downloads were selected in China and the United States (Sleep Cycle, Pillow, Sleep++, AutoSleep, and Calm in the 2 US app stores, and Little Sleep, Snail Sleep, Tidal Sleep, Sleep-White Noise, and Sleep Cycle in the 4 Chinese app stores). Detailed information on the 10 selected apps is shown in Table 1.

**Table 1:** Overview of the apps included in the study.

App^a^	Mean app rating^b^, mean (SD)	Number of ratings or reviews^c^	Number of downloads^d^	Developer	Category
Sleep Cycle (United States)	4.4 (0.3)	>370,000	>50 million	Sleep Cycle AB	Health and fitness
Pillow	4.2 (0.2)	>60,000	>60 million	Neybox Digital Ltd	Health and fitness
Sleep++	3.6 (1.3)	>20,000	>40 million	Cross Forward Consulting, LLC	Health and fitness
AutoSleep	4.6 (0.2)	>170,000	>50 million	Tantsussa Holdings Pty Ltd	Health and fitness
Calm	4.3 (0.3)	>350,000	>90 million	Calm.com, Ine	Health and fitness
Little Sleep	4.7 (0.2)	>50,000	>50 million	XinChao Technology Co, Ltd	Health and fitness
Snail Sleep	4.0 (0.2)	>80,000	>90 million	Seblong Technology (Beijing) Co, Ltd	Health and fitness
Tidal Sleep	4.4 (0.1)	>100,000	>40 million	Guangzhou Moreless Network Technology Co, Ltd	Health and fitness
Sleep-White Noise	3.8 (1.0)	>20,000	>40 million	SeekerTech Co, Ltd	Health and fitness
Sleep Cycle (China)	3.5 (0.9)	>90,000	>70 million	Sleep Cycle AB	Health and fitness

^a^End date: March 31, 2022.

^b^The mean of mean user rating of the app in all selected mobile app stores.

^c^The total number of user rating or review times of the app in all selected mobile app stores.

^d^The total number of user download times of the app in all selected mobile app stores.

#### Crawling User-Generated Reviews From App Stores in China and the United States

The app stores provide user ratings and comment functions that allow users to rate and textually describe their experiences with the app. We used the Crawler and Qimai app data analysis platform [21] to obtain all user reviews published between March 31, 2018, and March 31, 2022, which contained user comments and ratings of the selected apps. A total of 488,071 user reviews were collected, including 267,589 (54.8%) American user reviews and 220,482 (45.2%) Chinese user reviews.

### Pipelines of Processes to Clean Review Texts Using Natural Language Processing Techniques

**Figure 1:**
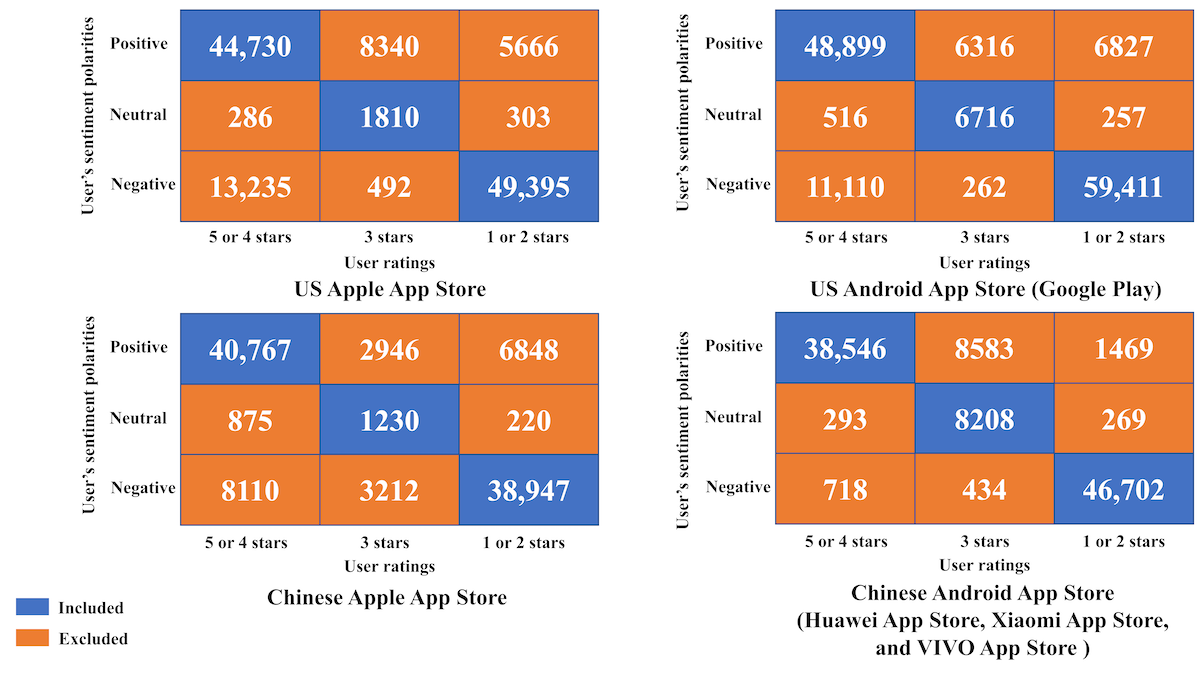
The result of sentiment analysis for preprocessing.

In the data preprocessing section, we applied the Python Natural Language Processing Toolkit and Sentiment Knowledge Enhanced Pretraining (SKEP) algorithm to preprocess the user review data of the 2 countries in the following seven steps:

1. Eliminated user reviews in languages other than English and Chinese.
2. Contradictory data with inconsistent user ratings and reviews were removed. We used the SKEP algorithm (its accuracy in the sentence-level sentiment classification task was 97.6%) [22] to judge the sentiment polarity of user reviews, which was compared with the ratings. When the ratings were 4 or 5 points, the sentiment polarity of the reviews was considered positive. When the ratings were 1 or 2 points, the sentiment polarity of the reviews was considered negative. On the basis of this assumption, we excluded mismatched data between the user reviews and ratings (Figure 1). Consequently, 87,587 mismatched user review data were excluded.
3. User reviews content text tokenization, then eliminated the numbers and punctuation marks of the content.
4. Language stop words (for English reviews using the System for the Mechanical Analysis and Retrieval of Text list and for Chinese reviews using the Harbin Institute of Technology list) and context-specific stop words such as the name of the mHealth sleep app were excluded.
5. The remaining words were filtered, retaining only the adverbs, adjectives, and nouns. Studies have shown that these words contain information about the product and product quality [23].
6. For English reviews, we stemmed and lemmatized each word to derive groups with the same root form.
7. Eliminated the data of blank records.

Finally, 372,730 user reviews were included after data preprocessing, including 202,963 (54.45%) American user reviews and 169,767 (45.55%) Chinese user reviews.

### Latent Dirichlet Allocation to Estimate the Number of Topics

We used latent Dirichlet allocation (LDA), a 3-level hierarchical Bayesian model in which each item of a collection is modeled as a finite mixture over an underlying set of topics, for collections of discrete data such as text corpora, which provides easy operation and high performance [24,25]. We used Python Gensim library to implement the LDA modeling. According to the perplexity curves, coherence scores, classification results, and realistic considerations (Figures S1-S6 in Multimedia Appendix 1), we identified 14 topics in Chinese and American user reviews.

### Tobit Model and Wald Test for Statistical Analyses Based on the Two-Factor Theory

The two-factor theory is the most widely replicated study on user satisfaction [17]. We set 2 dependent variables, namely, positive deviation (PD) and negative deviation (ND) [26], for data analysis. The 2 variables were defined by the difference between the user rating and the overall rating of the app in the app store. The range of values for PD was (0,4) and that for ND was (−4,0). The independent variable in this study was the probability distribution of each topic returned by the LDA model for each user review. In our study, the Tobit model, which is designed to estimate linear relationships between variables when there is either left-censoring or right-censoring in the dependent variable, was chosen to explore the factors influencing user satisfaction and dissatisfaction as follows:




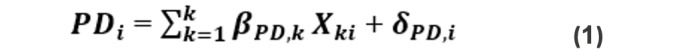







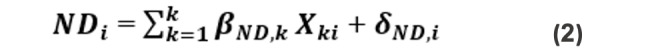




Where *β_k_* represents the correlation coefficient between the *k^th^* topic *X_ki_* for comment I and user satisfaction (*PD_i_* and *ND_i_*), k represents the number of topics included in the model, and *δ_i_* is the error term.

Finally, we applied the Wald test [26] to verify the variance of the parameters in the PD and ND models to analyze the asymmetrical effects of the factors influencing user satisfaction and dissatisfaction.

## Results

### Numbers of Topics and Impact Factors

A total of 488,071 user reviews of 10 mainstream mHealth sleep apps were obtained, and after data preprocessing, 372,730 (76.4%) user reviews (202,963, 54.5% from the United States and 169,767, 45.5% from China) were included. After LDA topic modeling, we identified 14 topics in Chinese and American user reviews (the topics identified in both sets were not identical). The topic modeling results are shown in Table 2 and Table 3.

**Table 2:** Topics and keywords of Chinese user reviews formulated by latent Dirichlet allocation topic modeling (N=169,767).

Topics	Keywords	Reviews, n (%)
Topic 1: cost	Free, price, sleep, pay, money, worth, and purchase	28,691 (16.9)
Topic 2: reliability	Update, issue, problem, cannot open, trash, fix, and disappoint	28,012 (16.5)
Topic 3: usability	Use, easy, help, daily, love, focus, and operate	22,748 (13.4)
Topic 4: sleep tracking function	Sleep, night, wake, track, pattern, asleep, and awake	18,165 (10.7)
Topic 5: sleep improvement effect	Sleep, feature, well, helpful, health, user, and improve	15,279 (9.0)
Topic 6: sleep advice function	Guide, voice, download, advice, read, review, and result	10,186 (6.0)
Topic 7: alarm function	Alarm, wake, morning, time, clock, start, and in bed	7130 (4.2)
Topic 8: attitude (positive)	Love, great, good, amaze, perfect, excellent, and tool	6960 (4.1)
Topic 9: compatibility	Watch, bracelet, Pad, support， not synchronize, sport, and trash	6621 (3.9)
Topic 10: sleep evaluation function	Analysis, evaluate, chart, average, record, sleep, hour, and accurate	5941 (3.5)
Topic 11: user interface	Awesome, interface, reminder, easy, find, complex, and share	5602 (3.3)
Topic 12: sound record function	Sound, snore, noise, sleep, record, interesting, and amazing	5093 (3.0)
Topic 13: advertisement distribution	Advertisement, game, videos, compulsion, annoying, and funny	4753 (2.8)
Topic 14: reminder function	Recommend, cycle, highly, world, anxious, person, and reminder	4586 (2.7)

**Table 3:** Topics and keywords of American user reviews formulated by latent Dirichlet allocation topic modeling (N=202,963)

Topics	Keywords	Reviews, n (%)
Topic 1: sleep tracking function	Sleep, night, wake, cycle, pattern, track, and graph	50,334 (24.8)
Topic 2: sound record function	Sound, record, support, hear, snore, noise, and issue	21,717 (10.7)
Topic 3: sleep improvement effect	Sleep, great, helpful, asleep, feeling, super, and everyday	20,702 (10.2)
Topic 4: user interface	Friendly, interface, user, pleasant, forward, improve, and share	18,064 (8.9)
Topic 5: usability	Easy, make, feel, nice, use, simple, and difference	14,004 (6.9)
Topic 6: alarm function	Wake, alarm, morning, clock, refresh, snooze, sound, and awake	12,786 (6.3)
Topic 7: sleep evaluation function	Sleep, review, give, star, change, download, and start	12,583 (6.2)
Topic 8: meditation function	Calm, sound, meditation, help, music, use, and daily	9539 (4.7)
Topic 9: reliability	Update, fix, version, effective, issue, open, and problem	9133 (4.5)
Topic 10: sharing function	Recommend, share, amaze, highly, friend, interest, and pretty	8727 (4.3)
Topic 11: sleep advice function	Love, advice, song, amaze, favorite, guide, and stay	7306 (3.6)
Topic 12: activity tracking function	Tone, track, perfectly, activity, move, body, and sleep	6900 (3.4)
Topic 13: cost	Worth, free, pay, money, price, subscription, and purchase	6697 (3.3)
Topic 14: compatibility	iPod, phase, watch, touch, science, trend, and nightly	4971 (2.4)

### Motivation and Hygiene Factors via Tobit Analysis

Tables 4 and 5 show the results of the Tobit analysis. Topic 8 in the Chinese data was user attitude (positive), and it was excluded from the Tobit model because it did not include user opinions on app features and effectiveness.

**Table 4:** Determinant factors for rating deviations (Chinese users).

Variable	Model 1: positive rating deviations^a^				Model 2: negative rating deviations^b^		
	Coefficient	SE	*P* value	Coefficient		SE	*P* value
Topic 1: cost	−1.232	0.025	<.001	−2.136		0.089	<.001
Topic 2: reliability	−0.909	0.022	.03	−3.268		0.096	.02
Topic 3: usability	−0.415	0.021	.007	−0.719		0.099	.002
Topic 4: sleep tracking function	−0.053	0.023	<.001	−0.226		0.098	<.001
Topic 5: sleep improvement effect	0.924	0.026	.005	1.185		0.131	<.001
Topic 6: sleep advice function	0.219	0.024	<.001	0.840		0.095	<.001
Topic 7: alarm function	0.541	0.026	.004	0.253		0.086	.003
Topic 9: compatibility	−1.125	0.024	<.001	−1.196		0.093	<.001
Topic 10: sleep evaluation function	0.094	0.025	<.001	0.612		0.096	<.001
Topic 11: user interface	−0.862	0.030	<.001	−0.429		0.092	<.001
Topic 12: sound record function	1.026	0.025	.004	0.098		0.108	<.001
Topic 13: advertisement distribution	−0.862	0.031	.004	−1.028		0.095	<.001
Topic 14: reminder function	0.862	0.030	.002	0.198		0.089	.004

^a^The maximum likelihood of model 1 was −46,902.561.

^b^The maximum likelihood of model 2 was −27,209.943.

**Table 5:** Determinant factors for rating deviations (American users).

Variable	Model 3: positive rating deviations^a^				Model 4: negative rating deviations^b^		
	Coefficient	SE	*P* value	Coefficient		SE	*P* value
Topic 1: sleep tracking function	−0.128	0.014	.002	−1.446		0.040	<.001
Topic 2: sound record function	−0.243	0.016	.003	−1.025		0.040	.002
Topic 3: sleep improvement effect	1.168	0.020	<.001	1.389		0.053	<.001
Topic 4: user interface	0.011	0.017	<.001	0.149		0.047	<.001
Topic 5: usability	0.861	0.018	<.001	0.346		0.050	<.001
Topic 6: alarm function	−0.049	0.015	.007	−0.305		0.046	.004
Topic 7: sleep evaluation function	−0.233	0.018	.004	−1.105		0.041	.006
Topic 8: meditation function	0.297	0.014	<.001	0.390		0.043	<.001
Topic 9: reliability	−0.826	0.018	<.001	−0.222		0.040	<.001
Topic 10: sharing function	0.054	0.016	.004	0.277		0.052	<.001
Topic 11: sleep advice function	0.197	0.016	<.001	0.444		0.059	<.001
Topic 12: activity tracking function	−0.296	0.018	.006	−0.518		0.048	<.001
Topic 13: cost	−0.426	0.016	<.001	−0.983		0.039	<.001
Topic 14: compatibility	−0.168	0.015	.006	−0.276		0.040	<.001

^a^The maximum likelihood of model 3 was −70,456.368.

^b^The maximum likelihood of model 4 was −29,095.359.

In Table 4, model 1 shows the results of the PD for the Chinese user data. We found that 13 user-discussed topics had a significant effect on the PD of user satisfaction. The variable with the largest positive effect was the app’s sound recording function (β=1.026; *P*<.001), and the variable with the largest negative effect was the value of money (β=−1.232; *P*<.001). Model 2 provides the results of ND for Chinese user data, and all 13 topics were statistically significant. The variable with the largest positive effect was the sleep improvement effect of the app (β=1.185; *P*<.001) and that with the largest negative effect was the reliability of the app (β=−3.268; *P*<.001).

In Table 5, model 3 shows the PD results for American user data. All 14 topics had a significant effect on the PD of user satisfaction; the variable with the largest positive effect was the app’s sleep improvement effect (β=1.168; *P*<.001) and that with the largest negative effect was the app’s reliability (β=−0.826; *P*<.001). Model 4 presents the results of the ND for American user data. All 14 topics included were statistically significant; the variable with the largest positive effect was the app’s sleep promotion effect (β=1.389; *P*<.001) and that with the largest negative effect was the sleep tracking function (β=−1.446; *P*<.001).

### Effects of Asymmetrical Attributes by Wald Testing

To explore the effects of asymmetry of the influencing factors, we used the Wald test to verify the differences in the parameters between models 1 and 2 and between models 3 and 4. The results of the Wald test are presented in Tables 6 and 7. We found that between models 1 and 2 and between models 3 and 4, all effect parameters were significantly different.

In Table 6, for Chinese users, 6 topics, namely, sleep improvement effect, sleep advice, alarm, sleep evaluation, sound record, and reminder function of the app, had a significant positive impact on the 2 models, with a significant difference in impact. In contrast, 7 topics, namely, the value of money, reliability, usability, sleep tracking, other device support, user interface, and advertisement, had a significant negative impact on both models.

In Table 7, for American users, 6 topics, namely, sleep improvement effect, user interface, ease of use, meditation, sharing function, and sleep advice, had significant positive effects on the 2 models, and the effects were significantly different. In contrast, 8 topics, namely, sleep tracking, sound recording, alarm, sleep evaluation, activity tracking, value of money, compatibility, and reliability, had a significant negative impact on both models, and the impact was significantly different.

**Table 6:** Comparison between the parameters of models 1 and 2 (Wald test).

Variable^a^	Wald test	*P* value	Reviews in PD^b^, n (%)	Reviews in ND^c^, n (%)
Topic 1: cost	3181.81	.004	4137 (14.42)	24,554 (85.58)
Topic 2: reliability	563.20	.03	9854 (35.18)	18,158 (64.82)
Topic 3: usability	1652.26	<.001	9251 (40.67)	13,497 (59.33)
Topic 4: sleep tracking function	8926.87	<.001	7956 (43.80)	10,209 (56.20)
Topic 5: sleep improvement effect	550.71	.008	9609 (62.89)	5670 (37.11)
Topic 6: sleep advice function	571.94	<.001	5727 (56.23)	4459 (43.77)
Topic 7: alarm function	148.67	.02	4333 (60.78)	2797 (39.22)
Topic 9: compatibility	594.64	<.001	1383 (20.89)	5238 (79.11)
Topic 10: sleep evaluation function	186.15	<.001	3143 (52.90)	2798 (47.10)
Topic 11: user interface	244.65	.04	1611 (28.76)	3991 (71.24)
Topic 12: sound record function	2937.53	<.001	3889 (76.37)	1204 (23.63)
Topic 13: advertisement distribution	783.92	.04	1436 (30.22)	3317 (69.78)
Topic 14: reminder function	284.35	<.001	2839 (61.92)	1747 (38.08)

^a^The total number of reviews for each topic: topic 1: 28,691, topic 2: 28,012, topic 3: 22,748, topic 4: 18,165, topic 5: 15,279, topic 6: 10,186, topic 7: 7130, topic 8: 6960, topic 9: 6621, topic 10: 5941, topic 11: 5602, topic 12: 5093, topic 13: 4753, and topic 14: 4586.

^b^PD: positive deviation.

^c^ND: negative deviation.

**Table 7:** Comparison between the parameters of models 3 and 4 (Wald test).

Variable^a^	Wald test	*P* value	Reviews in PD^b^, n (%)	Reviews in ND^c^, n (%)
Topic 1: sleep tracking function	2738.40	<.001	10,967 (21.79)	39,367 (78.21)
Topic 2: sound record function	826.85	.005	8072 (37.17)	13,645 (62.83)
Topic 3: sleep improvement effect	26.17	.03	19,244 (92.96)	1458 (7.04)
Topic 4: user interface	550.71	<.001	9868 (54.63)	8196 (45.37)
Topic 5: usability	2532.80	.002	11,004 (78.58)	3000 (21.42)
Topic 6: alarm function	102.38	<.001	4298 (33.62)	8488 (66.38)
Topic 7: sleep evaluation function	729.84	<.001	3698 (29.39)	8885 (70.61)
Topic 8: meditation function	129.39	.04	5742 (60.20)	3797 (39.80)
Topic 9: reliability	134.82	<.001	3918 (42.90)	5215 (57.10)
Topic 10: sharing function	2738.98	<.001	4801 (55.02)	3926 (44.98)
Topic 11: sleep advice function	7389.03	.01	4653 (63.70)	2653 (36.30)
Topic 12: activity tracking function	182.04	<.001	2887 (41.85)	4013 (58.15)
Topic 13: cost	249.94	.004	1869 (27.91)	4828 (72.09)
Topic 14: compatibility	1049.89	<.001	1532 (30.82)	3439 (69.18)

^a^The total number of reviews for each topic: topic 1: 50,334, topic 2: 21,717, topic 3: 20,702, topic 4: 18,064, topic 5: 14,004, topic 6: 12,786, topic 7: 12,583, topic 8: 9539, topic 9: 9133, topic 10: 8727, topic 11: 7306, topic 12: 6900, topic 13: 6697, and topic 14: 4971.

^b^PD: positive deviation.

^c^ND: negative deviation.

## Discussion

### Principal Findings

#### Satisfaction of Chinese and American Users With mHealth Sleep Apps

The overall user satisfaction rate of mHealth sleep apps was poor, and compared with the US users, Chinese users were slightly more satisfied with such apps. Among the Chinese user review data, 96,056 user reviews had ratings higher than 3, indicating a user satisfaction rate of 56.58%. Among the US user review data, 93,094 reviews had ratings higher than 3, indicating a user satisfaction rate of 45.87%. The low user satisfaction rates explain the poor user engagement and low willingness to use mHealth sleep apps [11]. However, this result is inconsistent with the findings of some user satisfaction studies using small samples [14-16], such as those by Philip et al [16], which indicated that 91.6% (395/431) of users rated the system as “satisfactory” or above and 61.7% (266/431) rated the system as “very satisfactory.”

Users in both countries commented on the app’s functionality, usability, reliability, compatibility, user interface, value of money, and sleep improvement effects. First, users discussed the functions of the apps, mainly focusing on sleep monitoring, sound and sleep movement recording, and the user experience related to these features. Second, users discussed the usability and compatibility of the apps, focusing on the various failures encountered during use, such as flashbacks and lagging. They also provided feedback on app compatibility with other devices (sports bracelets, sports watches, etc), such as information that was not synchronized or incompatible. Third, feedback on the user interface of the app mainly focused on the design, user experience after interacting with the interface, and distribution of advertisements. The use of too many advertisements was a common source of dissatisfaction. Moreover, users heavily discussed the apps’ value for money, mainly concerning the price of the app and charging issues, which led to user dissatisfaction. Finally, the effect of the app’s intervention on sleep was another aspect of concern.

According to the probability of topic distribution, we found that the content of discussions about mHealth sleep apps differed significantly between the Chinese and American users. American users cared more about the functions of the apps, discussing sleep tracking (50,334/202,963, 24.8%) and sound recording functions (21,717/202,963, 10.7%) significantly more than the value for money (6697/202,963, 3.3%) and compatibility (4971/202,963, 2.25%). American users also mentioned the user interface design of the app (18,064/202,963, 8.9%). In contrast, Chinese users were price sensitive and discussed the app’s cost (28,691/169,767, 16.9%), reliability (28,012/169,767, 16.5%), and usability (22,748/169,767, 13.4%).

#### Hygiene and Motivation Factors of mHealth Sleep Apps

The results of the Wald test demonstrated that all effect parameters in the 4 models were significantly different, indicating that the factors influencing Chinese and American user satisfaction with mHealth sleep apps were asymmetrical. Therefore, we obtained the hygiene and motivation factors of mHealth sleep apps in both countries.

We found that the cost, reliability, usability, compatibility, user interface, advertisements, and sleep monitoring functions of the app were the hygiene factors of Chinese mHealth sleep apps. The hygiene factors for American mHealth sleep apps included reliability, cost, and compatibility of the app; sleep monitoring; sound and sleep movement recording; smart alarm; and sleep assessment functions. The probability of the above topics appearing in user reviews increased with the degree of user dissatisfaction, indicating that the above factors were the basic user-expected attributes of sleep apps. The sleep promotion effect, sleep suggestion, smart alarm, sleep assessment, sleep sound recording, and reminder functions of the app were the motivation factors for Chinese mHealth sleep apps. The motivation factors of American sleep apps included the sleep promoting effect, user interface, usability, meditation, sleep suggestion, and sharing functions of the app. The probability of the above features appearing in user reviews positively influenced user satisfaction, indicating that the above factors were the value-added attributes of sleep apps.

Using the China-US comparison, we found that user satisfaction was mostly related to the app’s sleep promotion effects and sleep advice function, whereas user dissatisfaction was mostly related to the app’s stability, compatibility, value of money, and sleep tracking function.

#### Suggestions for Improving Users’ Intentions to Continue the Use of mHealth Sleep Apps

An in-depth analysis of the asymmetry of factors influencing user satisfaction and dissatisfaction with mHealth sleep apps is of great value in providing a reference for improving users’ intentions to continue using mHealth sleep apps.

As a strategy for promoting the continued use intentions of sleep app users, hygiene factors should be improved. First, the reliability and usability of the app should be improved, as studies have shown that poor usability is one of the most common reasons for users to abandon mHealth apps [27]. Second, experts need to enhance the compatibility of apps with wearable devices and solve problems such as unsynchronized terminal data app pricing. Finally, sleep apps should provide more accurate sleep tracking services. Although sleep monitoring is the core function of mHealth sleep apps, users are generally dissatisfied with the accuracy of this function. Under the premise of improving hygiene factors, experts should focus on the sleep promotion effect and sleep advice function of apps to further improve user satisfaction. Scientific and effective sleep advice is the basis of sleep promotion, which is the core goal of mHealth sleep apps, but studies have shown that existing apps lack clinical validation of sleep promotion effects [28]. Therefore, experts should conduct clinical trials of different scales to fully validate the effectiveness of apps in sleep intervention while continuously improving the internal app algorithms to provide more effective sleep recommendation services based on scientific evidence.

### Limitations

This study had several limitations. First, because not all users provide reviews, we cannot evaluate the satisfaction with mHealth sleep apps of such users, which could lead to selection bias. Nevertheless, 372,730 user reviews were analyzed, which provided sufficient data to explore user satisfaction with mHealth sleep apps and their influencing factors. Second, our analysis did not consider the characteristics of app users and the metadata of user reviews, which may have improved the accuracy of the model and significantly affected the results. However, the user review data in mobile app stores usually contain the user’s name, release date, user score, and user text reviews. In addition, we cannot obtain the user characteristics and metadata of user reviews without the assistance of app developers. This is a common limitation in user-generated content studies. Moreover, only user reviews of 10 mainstream mHealth sleep apps from mobile app stores in China and the United States were included, leading to possible bias. However, China and the United States have the highest number of internet users in the world, and the number of active users of mainstream apps far exceeds that of other apps. Thus, we believe that the results of this study are representative and adequate to reflect the overall satisfaction with mHealth sleep apps.

### Conclusions

To improve users’ intention to continue using mHealth sleep apps, we identified motivation and hygiene factors for such app use by processing and comparing >480,000 user reviews of 10 mainstream sleep apps in China and the United States. We found that the user satisfaction rates of mHealth sleep apps were poor, resulting in users’ low continued use intentions. Moreover, the effects of the influencing factors of Chinese and American user satisfaction and dissatisfaction with mHealth sleep apps were asymmetrical. Overall, the motivation factors for mHealth sleep apps were the sleep suggestion function and sleep promotion effects of the apps. The hygiene factors included the reliability, compatibility, value of money, and sleep monitoring function of the apps. To promote the use of mHealth sleep apps in the real world, we should first improve the hygiene factors and then achieve the motivation factors. In future research, we should focus on the characteristics of the user and the metadata of user reviews and then introduce them into the equation as covariates to further improve the accuracy of the model.

## Appendix

Multimedia Appendix 1 Supplementary figures.

## Abbreviations

LDA: latent Dirichlet allocation
mHealth: mobile health
ND: negative deviation
PD: positive deviation
SKEP: Sentiment Knowledge Enhanced Pretraining

## Data Availability

The user review data were extracted between March 31, 2018, and March 31, 2022, through the Crawler and the Qimai app data analysis platform from 4 Chinese mobile app stores (China Apple App Store, Huawei App Store, Xiaomi App Store, and VIVO App Store) and 2 mobile app stores in the United States (US Apple App Store and US Android Google Play Store). The source code for our analysis is publicly available at GitHub [29].
