# Supplementary material for: Mining the Influencing Factors and Their Asymmetrical Effects of mHealth Sleep App User Satisfaction From Real-world User-Generated Reviews: Content Analysis and Topic Modeling
Source: J Med Internet Res. 2023 Jan 31;25:e42856. doi: 10.2196/42856 (PMC9929723; doi:10.2196/42856)
Supplement: Multimedia Appendix 1 [file jmir_v25i1e42856_app1.docx]

**supplementary material**

**Table of Contents:**

[Fig. S1. Topics coherence score (rang between 2 and 29 topics) of Chinese user reviews ２](#_Toc1253812710)

[Fig. S2. Topics perplexity score (rang between 2 and 29 topics) of Chinese user reviews ３](#_Toc1720672606)

[Fig. S3. Topics coherence score (rang between 2 and 29 topics) of American user reviews ４](#_Toc1329698540)

[Fig. S4. Topics perplexity score (rang between 2 and 29 topics) of American user reviews ５](#_Toc1528531098)

[Fig. S5. Overview of the formulation of 14 topics in Chinese user reviews with LDA topic modeling ６](#_Toc1822778672)

[Fig. S6. Overview of the formulation of 14 topics in American user reviews with LDA topic modeling ７](#_Toc1586915849)

**
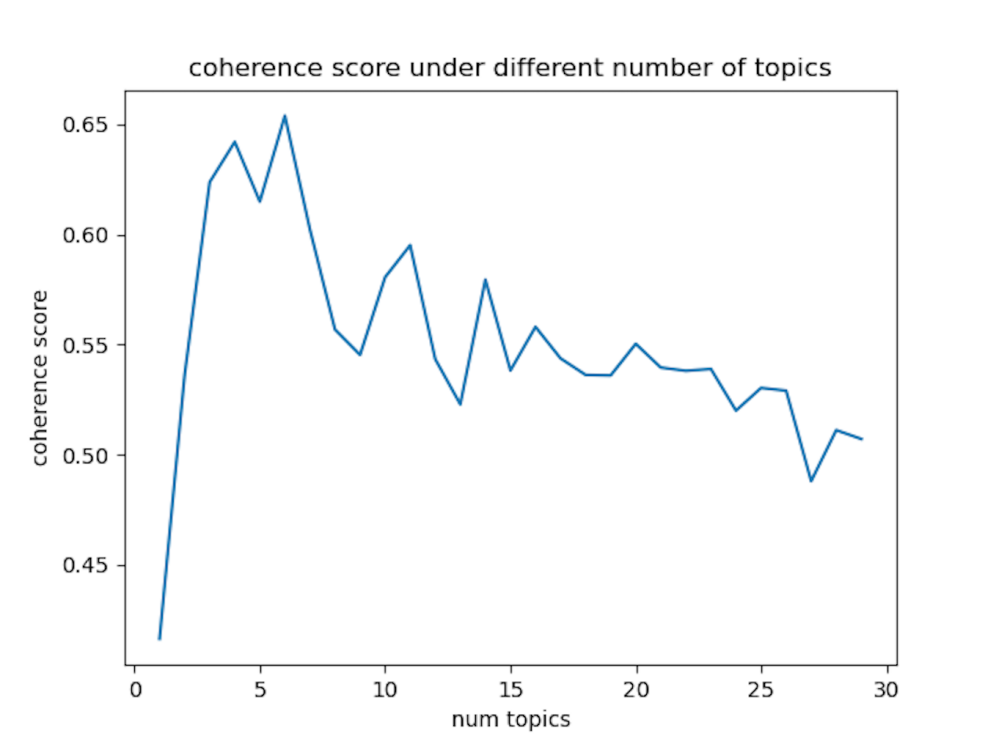
**

# Fig. S1. Topics coherence score (rang between 2 and 29 topics) of Chinese user reviews

**
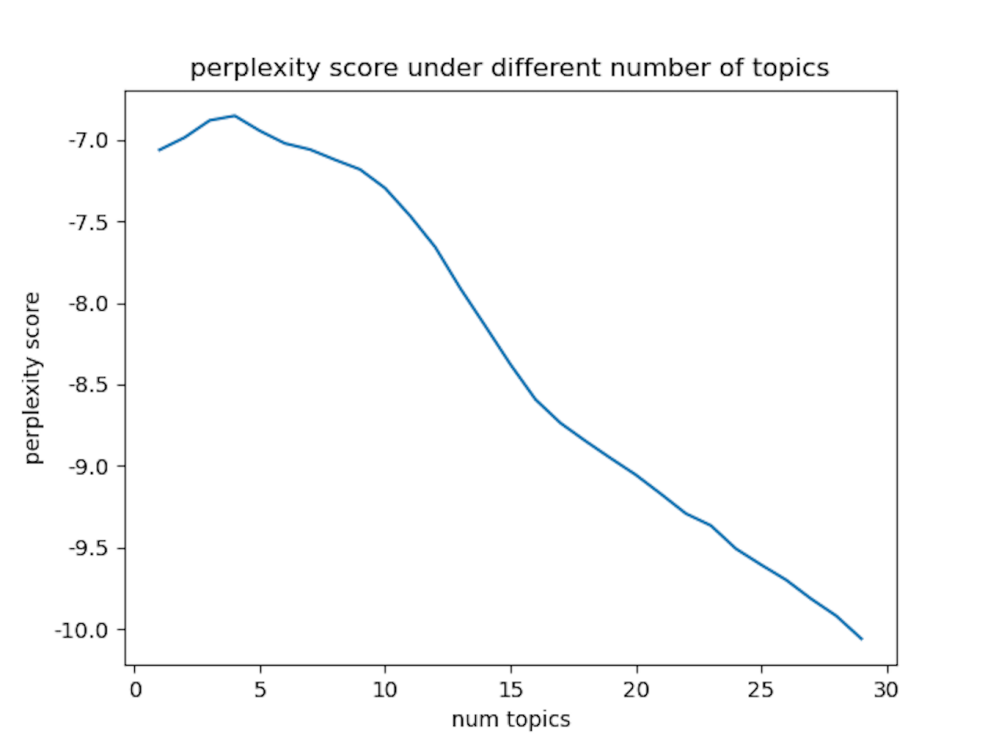
**

# Fig. S2. Topics perplexity score (rang between 2 and 29 topics) of Chinese user reviews

**
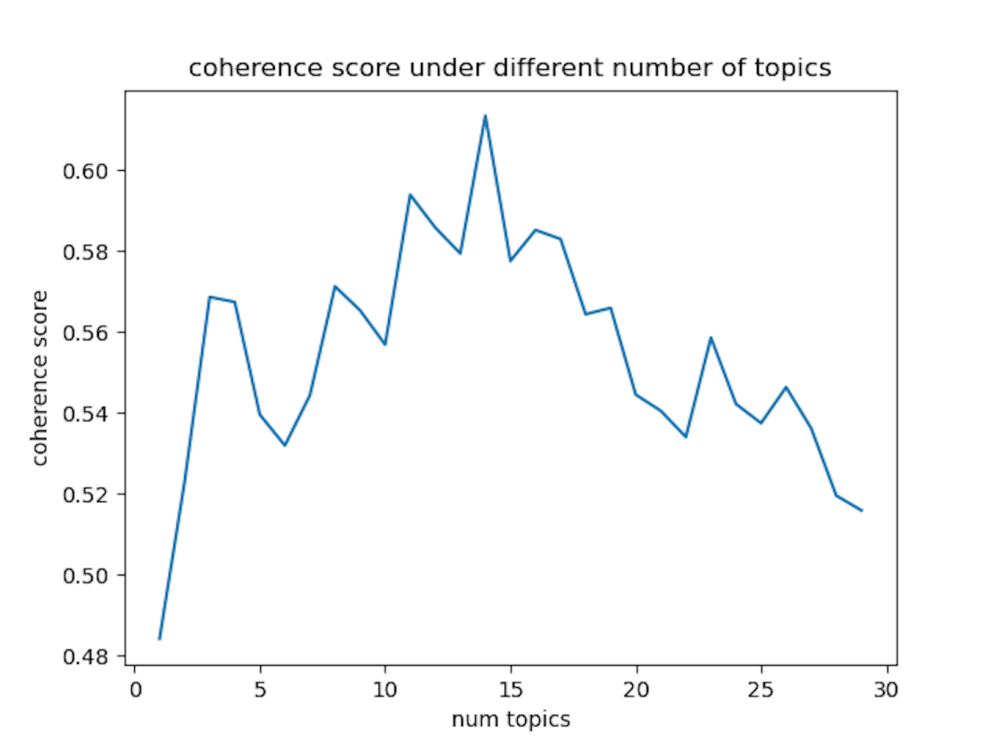
**

# Fig. S3. Topics coherence score (rang between 2 and 29 topics) of American user reviews

**
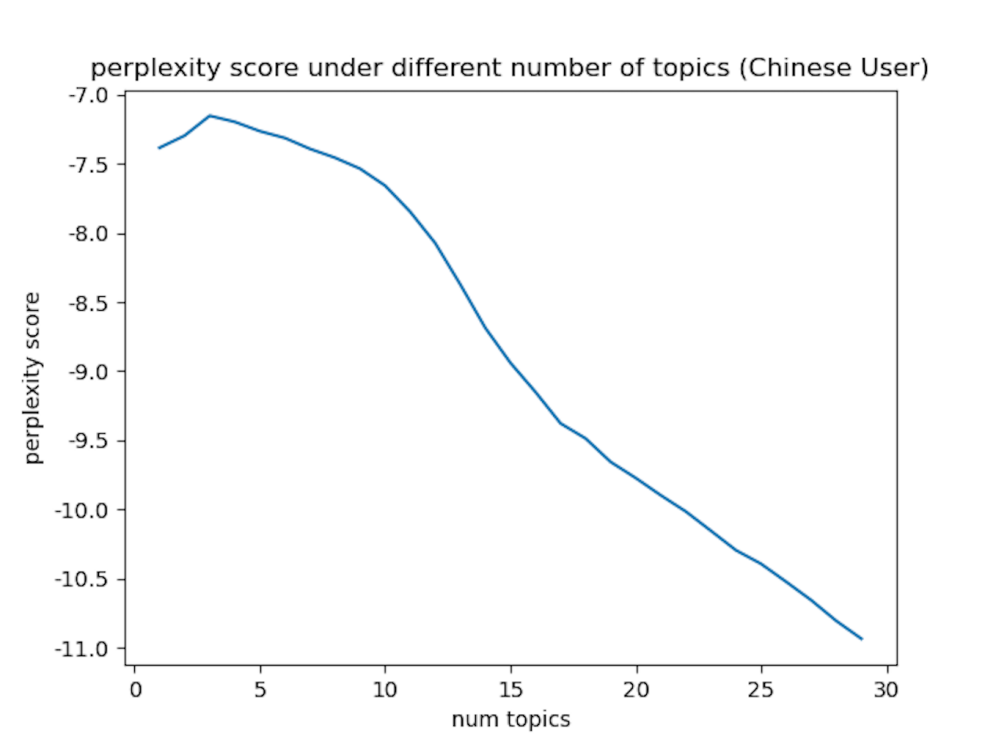
**

# Fig. S4. Topics perplexity score (rang between 2 and 29 topics) of American user reviews

**
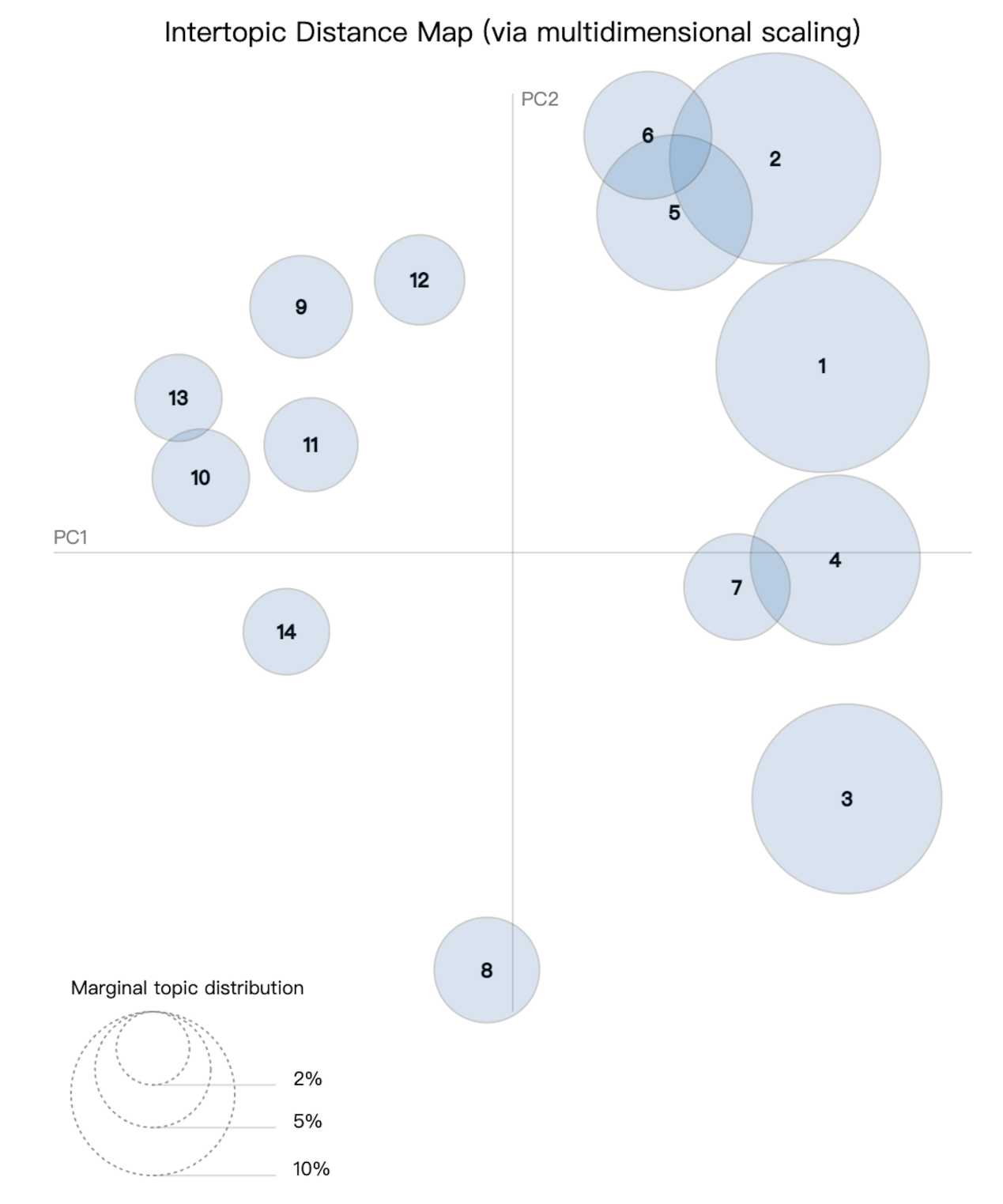
**

# Fig. S5. Overview of the formulation of 14 topics in Chinese user reviews with LDA topic modeling

**
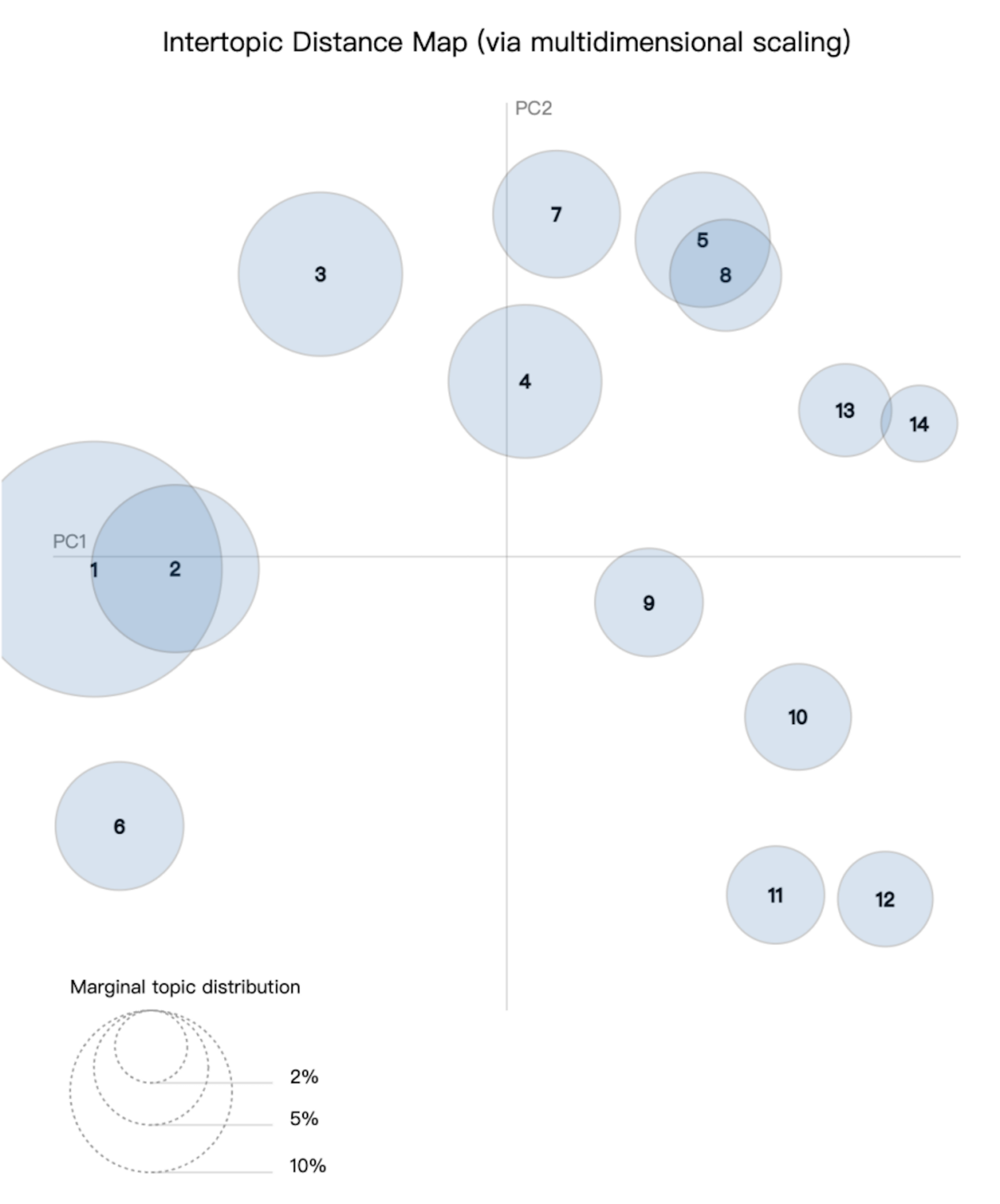
**

# Fig. S6. Overview of the formulation of 14 topics in American user reviews with LDA topic modeling
